# Current approaches to the surgical management of Crohn’s disease in Australia and New Zealand

**DOI:** 10.1007/s00384-024-04778-6

**Published:** 2025-01-03

**Authors:** Sophie Zheng, Aleksandra Edmundson, David A. Clark

**Affiliations:** 1 Royal Brisbane and Women’s Hospital, Butterfield St., Herston, QLD 4006 Australia; 2 Faculty of Medicine, University of Queensland, Brisbane, QLD Australia

**Keywords:** Crohn’s disease, Colorectal surgery, Surgical anastomosis, Colectomy

## Abstract

**Purpose:**

Given the evolving literature regarding the optimal surgical approach to mitigate post-operative recurrence of Crohn’s disease (CD), this survey study aimed to elucidate the practices and preferences of colorectal surgeons in Australia and New Zealand (ANZ) in their surgical management of CD.

**Methods:**

Colorectal surgical consultants and fellows (*n* = 337) registered with the Colorectal Surgical Society of Australia and New Zealand (CSSANZ) were invited by email in April 2022 to participate in a cross-sectional survey consisting of basic demographics and 12 questions relating to their usual surgical practice and preferred operative strategy.

**Results:**

A total of 135 responses were received (39.9%). Regarding anastomotic configuration, 47% (*n* = 68) preferred the side-to-side anastomosis (STSA), 19% (*n* = 28) the end-to-end anastomosis (ETEA), and 15% (*n* = 21) the Kono S anastomosis. Most respondents preferred to resect at the proximal junction of the abnormal mesentery (75%, *n* = 97), while radical resection of the mesentery was preferred in 10% (*n* = 13) and close intestinal resection through abnormal mesentery in 15% (*n* = 20). The preferred surgical approach was by far laparoscopic (93%, *n* = 125) with extraction from the midline peri-umbilical port (80%, *n* = 108).

**Conclusion:**

Amongst participating colorectal surgeons, there was a clear consensus on the approach, where the dominant practice was laparoscopy with a midline peri-umbilical extraction. Similarly, most respondents preferred some degree of mesenteric resection. However, anastomotic configuration and technique were domains of resection in CD lacking unanimity despite clear guidelines, highlighting an area requiring further attention.

**Supplementary Information:**

The online version contains supplementary material available at 10.1007/s00384-024-04778-6.

## Introduction

Crohn’s disease (CD) stands as a formidable challenge within the realm of gastrointestinal disorders, characterized by its chronic inflammation and unpredictable course. The burden it places on healthcare systems and society is substantial, with estimated yearly hospital costs of inflammatory bowel disease (IBD) totalling over $100 million in Australia, extending to over $380 million when considering productivity losses [1]. The prevalence of CD is estimated at 306 per 100,000 in Australia and New Zealand (ANZ) [2]. While medical therapy remains the cornerstone of management, up to 80% of CD patients ultimately require surgical intervention [3]. However, surgery seldom offers a cure, as 50% of CD patients face the reality of recurrent disease necessitating a second operation within 10 years [4]. Notably, rates of histological recurrence have been reported to be as high as 83–100% at 3 years and often the clinical course progresses from endoscopic post-operative recurrence (ePOR) to symptomatic recurrence requiring re-operation with time [5, 6]. These observations underscore the urgency in refining surgical techniques to reduce recurrence.

A 2014 meta-analysis supported the superiority of the stapled side-to-side anastomosis (STSA) over the handsewn end-to-end anastomosis (ETEA) in reducing rates of anastomotic leakage (AL) and postoperative endoscopic recurrence [7]. These findings are echoed in the European Crohn’s and Colitis Organisation (ECCO 2019) Guidelines for CD ileocolic resection (ICR) [8].

Developing insight into the role of the mesentery in CD pathogenesis has fuelled interest in surgical approaches that resect or isolate this putative key driver of recurrence [9–11].

The Kono S anastomosis, pioneered in 2003 and described by Professor Toru Kono et al. and reported in 2011, has garnered attention for its potential to prevent recurrence by separating the anastomosis from the mesentery [10, 12–14]. The anastomosis is a handsewn antimesenteric functional ETEA which creates a stricturoplasty-like anastomosis between longitudinal entero-colotomies, supported by a column of transverse staple lines from the stumps of the intestinal resections. This configuration functions to isolate the anastomosis from the preserved mesentery and may facilitate post-operative colonoscopy, an advantage of the ETEA over the STSA [7, 14, 15]. The Kono S anastomosis was studied in a randomised controlled trial (RCT) by Luglio et al., who found a statistically significant reduction in ePOR in favour of the Kono S (*p* < 0.001) [12].

Concurrently, Coffey et al. argues for the inclusion of the mesentery with ICR, finding significantly lower reoperation rates with mesenteric division rather than flush intestinal division (*p = *0.0003) [11]. Mesenteric excision and exclusion (MEE) techniques described by Holubar et al. in 2022 incorporate mesenteric resection into a modified Kono S anastomosis to further reduce recurrence, an approach demonstrated to be safe and feasible in practice [16].

In the setting of this ongoing debate surrounding the optimal surgical approach for CD, a survey has been developed and distributed to elucidate the prevailing practices and preferences of colorectal surgeons in Australia and New Zealand regarding anastomotic configuration and mesenteric resection. This study aims to inform future directions and research in the surgical management of CD and potentially pave the way for improved outcomes in this complex patient population.

## Methods

### Study design

A cross-sectional survey was administered to assess current practice amongst Australian and New Zealand colorectal surgeons in April 2022 (Appendix 1). The St Vincent’s Private Hospital Northside Human Research and Ethics Committee granted approval for the study protocol (No. HREC 18/21). Surgeon’s participation was voluntary with no coercion or reimbursement to complete the survey. Informed consent was confirmed at invitation, and the return of a completed survey indicated agreement to participate in the study. This was outlined in the requirement of participation.

### Participants

Surgical consultants and fellows (*n* = 337) registered with the Colorectal Surgical Society of Australia and New Zealand (CSSANZ) were invited by email to participate in this study online, requiring the completion of a short survey taking approximately 2 min to complete. All invited surgeons were assigned a unique three-digit code known to a third party (research administration of CSSANZ) administering the survey on behalf of the investigators. The invitation outlined the purpose of the survey and the requirements for participation.

### Study survey

The study survey consisted of basic demographics and 12 questions related to surgeons’ usual surgical practice and was constructed by the coordinating principal investigator. Demographic data captured included surgeons’ age, gender, CSSANZ membership status, location and regionality of the practice, and if their primary facility had a dedicated IBD service. The second section captured clinical information, including the number of ICRs for CD performed in 12 months, along with the surgeon’s preference for anastomotic configuration, technique, approach, extraction site, tissue margin, and bowel preparation. Surgeons were asked if they had performed the Kono S anastomosis, how they were studying outcomes, and if they were interested in participating in a RCT of the Kono S anastomosis (Appendix 1). The survey was distributed on April 13th 2022. Only one reminder email was sent by CSSANZ Research Support Committee, two weeks following the initial invitation. No validation methods were used for this survey design.

The investigators did not have access to the members’ personal details. All members of CSSANZ were deidentified by a third party by the assignment of a unique study code, unknown to the investigators. Members of CSSANZ received surveys electronically using REDCap, a password-protected data capture management system. Hard copies of surveys were also made available to participants who chose to complete surveys on paper.

### Statistical methods

GraphPad Prism ver. 9.0 (GraphPad, San Diego, CA, USA) and Microsoft Excel for Microsoft 365 (Version 2402) 32-bit were used for analysis. A *p* value of < 0.05 was considered significant. Associations with categorical variables were analysed by two-sided Fisher’s exact test or Chi-squared test. Ordinal variables were analysed by unpaired two-tailed *t*-test with Welch’s correction or an ordinary one-way ANOVA with Tukey’s multiple comparisons.

## Results

### Participant demographics

A total of 135 responses were received (39.9%). The survey was completed in its entirety by 131 of respondents. Demographics are summarised in Table 1.

**Table 1:** Participant demographics

Characteristic					*n*	%
Gender						
Male					114	84%
Female					21	16%
Member stats						
Consultant					112	83%
Fellow					23	17%
Region						
	Metropolitan		Regional		Total	
	*n*	%	*n*	%		
NSW	31	86%	5	14%	36	27%
NZ	23	85%	4	15%	27	16%
QLD	17	77%	5	23%	22	21%
SA	11	100%	0	0%	11	8%
TAS	1	33%	2	67%	3	2%
VIC	25	89%	3	11%	28	6%
WA	8	100%	0	0%	8	20%
Total	116	86%	19	14%		
Approximate number of ileocolic resections for Crohn’s disease per year			Average		6.8 ± 5.4	
			Median		5 (6.8)	
Dedicated IBD service at primary hospital					91	67%

Survey respondent demographics (*n* = 135). Values are expressed as *n*, %, mean ± SD, or median (IQR). *NSW*, New South Wales; *QLD*, Queensland; *VIC*, Victoria; *SA*, South Australia; *TAS*, Tasmania; *SA*, South Australia; *WA*, Western Australia; *NZ*, New Zealand; *IBD*, inflammatory bowel disease

Mean surgeon age was 47 ± 9.96 years. The mean age was higher for male surgeons (48) than for female surgeons (44), and the mean age of consultant surgeons was (48) higher than fellows (44) (Table 1). As no responses were received from the Northern Territory (NT), this region was not included.

On average, fellows performed 2.35 more resections than consultants (8.74 vs 6.39, *p* = 0.03, CI 0.23–4.46) and surgeons primarily based in metropolitan facilities performed an average of 3.07 more resections than their regional counterparts (7.22 vs 4.16; *p = *0.02, CI 0.46–5.67). The presence of a dedicated IBD service at the primary hospital is correlated with an increased mean number of yearly resections per surgeon by 3.82 (8.04 *vs* 4.22, *p *< 0.0001, CI 2.34–5.31).

### Anastomotic configuration

**Fig. 1:**
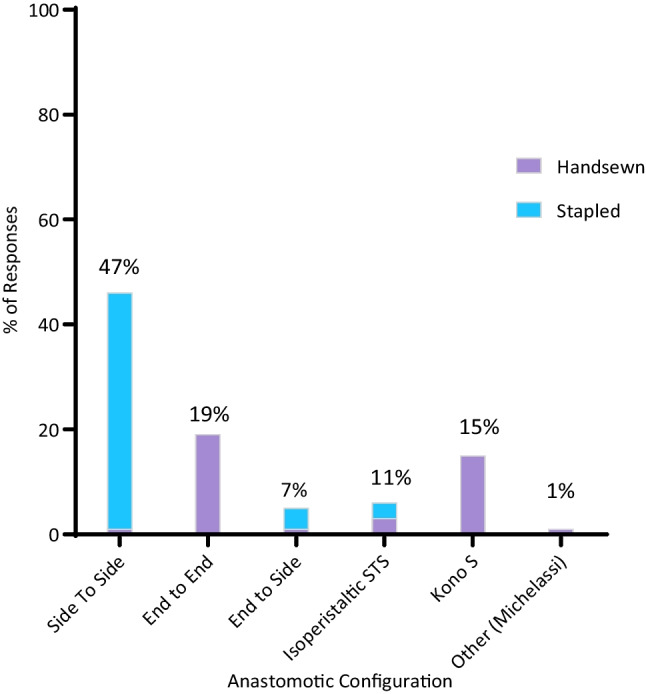
Preferred anastomotic configuration and technique after ileocolic resection for Crohn’s disease. % of responses on the *y*-axis. *n* = 144 responses. Data label denotes combined percentage of both handsewn and stapled technique for each anastomotic configuration. STS, side to side

Surgeon preference for anastomotic configuration for ICR is presented in Fig. 1. Seven respondents selected more than one option for this item, thus the total number of responses (*n* = 144) exceeds the number of survey respondents (*n* = 135). The STSA was the leading preferred anastomotic configuration (47%), followed by the ETEA (19%). Importantly, the Kono S was preferred by 15% of respondents. The sole respondent for “other” specified the Michelassi strictureplasty.

**Fig. 2:**
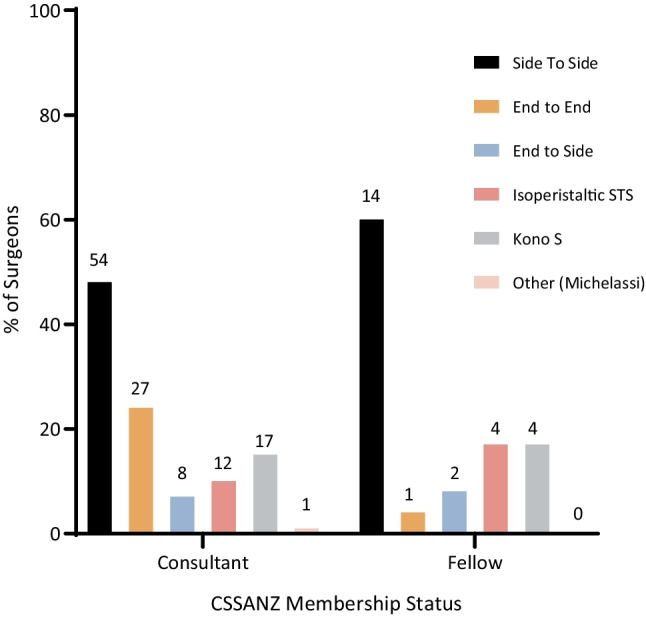
Comparison of distribution in anastomotic preference by status of membership in CSSANZ. % of responses within either consultant or fellow subgroup on the *y*-axis. *n* = 144 responses. The order of data bars from left to right corresponds to the descending order of data sets in the legend. The data label denotes *n* for each anastomotic configuration. STS, side to side

The preferred configuration was not affected by whether a surgeon’s primary hospital had a dedicated IBD service (*p* = 0.75). Comparisons for each configuration are summarised in Table 2. 93% of responses preferring the STSA preferred to perform a stapled anastomosis, whereas 100% of surgeons preferring the ETEA favoured handsewn. By nature of the technique, 100% of Kono S respondents preferred a handsewn anastomosis, which is in comparison to 20% for the ETSA and 31% for the isoperistaltic STSA. Overall, anastomoses were preferred to be stapled by 61% and handsewn by 38%. On average, more fellows preferred the stapled anastomosis than consultants, 76% vs 59% (*p* = 0.01). The distribution of preferred anastomotic configuration between consultant and fellow respondents was not significantly different (Fig. 2) (*p = *0.95). An IBD service had no significant effect on surgeon preference for a handsewn or stapled anastomosis, with 58% of surgeons with a dedicated service and 67% of those without preferring the stapled anastomosis (*p* = 0.35). Overall, the stapled STSA was the preferred anastomosis following resection for Crohn’s disease, preferred by 46% of respondents.

**Table 2:** Preferred anastomotic configuration of surgeons based in hospitals with and without a dedicated IBD service

	IBD service		No IBD service	
	*n*	(%)	*n*	(%)
Side to side	43	(47)	25	(47)
End to end	18	(20)	10	(19)
End to side	6	(6.7)	4	(7.5)
Isoperistaltic side to side	12	(13)	4	(7.5)
Kono S	11	(12)	10	(19)
Other (Michelassi)	1	(1.1)	0	(0)

*IBD*, inflammatory bowel disease. Values are given as % of column total and (*n*)

### Surgical approach

**Fig. 3:**
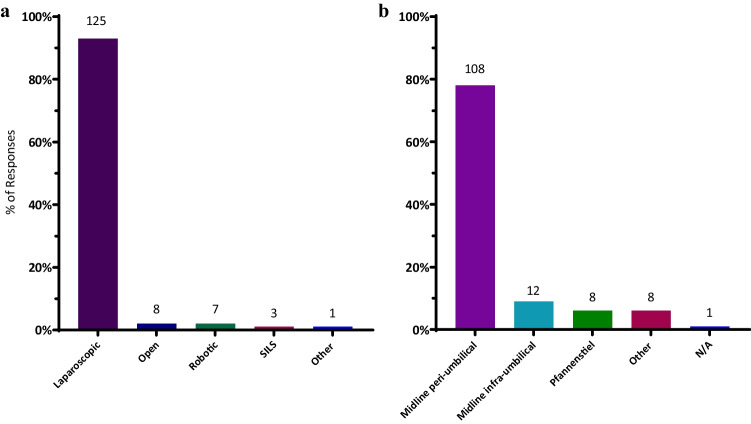
**a** Preferred approach to ileocolic resection. Percentage of respondents on the *y*-axis. *n* = 144. Data label denotes *n* of responses SILS, single incision laparoscopic surgery. **b** Preferred extraction site if the minimally invasive approach was preferred. Percentage of responses on the *y*-axis. *n* = 138. Data label denotes *n* of responses

The minimally invasive platform was the preferred approach. By far, the preferred approach was laparoscopic (93%). Only 6% of respondents preferred an open approach to resection. One participant responded “other”, preferring the single incision laparoscopic surgery (SILS) mobilisation (Fig. 3a).

Of the surgeons preferring a minimally invasive approach, the midline peri-umbilical port site was the preferred extraction site at 80% (Fig. 3b). Responses within the “other” category included the left iliac fossa, muscle sparing supra-umbilical oblique, right transverse, muscle splitting right iliac fossa, and transverse right upper quadrant. As with prior survey items, the total number of responses (*n* = 144) exceeds the number of survey respondents (*n* = 135). No significant difference was found for the preferred approach between consultant and fellow status (*p* = 0.36).

### Mesenteric resection

With respect to approaches to the mesentery, most respondents preferred to resect at the proximal junction of abnormal mesentery (75%, *n* = 97). Ten percent (*n* = 13) of respondents preferred a radical resection of the mesentery, and 15% (*n* = 20) preferred a close intestinal resection through abnormally thickened mesentery.

The average margin of normal tissue targeted for resection was 2.2 ± 1.5 cm. Consultant surgeons targeted a smaller mean tissue margin (2.1 ± 1.3 cm) than fellows (2.7 ± 2.2 cm), but the mean difference was not significant (*p = *0.19). There was no significant difference in the target tissue margin with respect to the extent of mesenteric resection (*p* = 0.69).

### Bowel preparation

A full pre-operative bowel preparation regime was preferred by 32% of respondents, and 37% preferred a fluid-only restriction. “Other” comprised 30% of respondents, and responses included a fleet enema, no preparation, oral antibiotics and carbohydrate loading, exclusive enteral nutrition, or a standard nil-by-mouth fast. As one respondent left this item unanswered, only 134 responses were received. Overall, there appears to be no consensus for pre-operative bowel preparation in instances of ICR for CD.

### Kono S anastomosis

Overall, 21% (*n* = 29) of respondents had performed the Kono S anastomosis (Table 3). Though a greater proportion of fellows (30%, 7 of 23) reported having performed a Kono S than consultants (20%, 22 of 112), this was not a statistically significant difference, *χ*^2^ (1, *N* = 29) = 1.04, *p = *0.31. The geographical distributions of respondents having performed the Kono S significantly varied from that of the overall respondents, with more surgeons from NSW, VIC, and SA having performed the Kono S, *χ*^2^ (6, *N* = 29) = 12.75, *p = *0.047 (Table 4).

**Table 3:** CSSANZ membership status of surgeons who had performed the Kono S anastomosis

	Expected		Observed	
	*n*	(%)	*n*	(%)
Consultant	24	83	22	76
Fellow	5	17	7	24
Total	29	100	29	100

Comparison of observed distribution vs expected distribution according to overall participant demographics

**Table 4:** Geographical distribution of surgeons who had performed the Kono S anastomosis

	Expected		Observed	
	*n*	(%)	*n*	(%)
NSW	8	27	14	48
QLD	5	16	1	3
VIC	6	21	7	24
SA	2	8	4	14
TAS	1	2	0	0
WA	2	6	1	3
NZ	6	20	2	7
Total	29	100	29	100

Comparison of observed distribution vs expected distribution according to overall participant demographics

Of surgeons practising primarily at a hospital with a dedicated IBD service, 26% had performed the Kono S anastomosis, compared to 11% of those primarily based at a facility without an IBD service. There was no significant difference in the adoption of the Kono S anastomosis in centres with or without an IBD service (*p* = 0.07). Of participants who had performed the Kono S anastomosis (*n* = 29), 45% were studying outcomes in a prospective audit, 24% in a retrospective cohort study, and 14% in a RCT. 49% of respondents were interested in participating in a multi-site randomised study of the Kono S anastomosis.

Of the respondents who preferred the Kono S anastomosis (*n* = 21), only 4 preferred to resect the abnormal mesentery flush against the intestine, whereas 15 preferred to resect the abnormal mesentery at its most proximal junction, and 2 preferred radial resection of the mesentery. Similarly, of all respondents who reported having performed the Kono S (*n* = 29), 5 preferred conservative, limited resection, 21 preferred resection at the proximal junction of abnormal mesentery, and 3 preferred radical resection.

## Discussion

In light of emerging evidence for the Kono S anastomotic configuration and the role of mesenteric resection on CD recurrence, this study aimed to examine current surgeon practices in managing CD using a single cross-sectional survey of colorectal surgeons practising in ANZ. The survey evaluated elements of preferred practice and approach to surgical resection in CD with respect to anastomotic configuration, technique, surgical approach, mesentery handling, target tissue margin, extraction site, and bowel preparation.

Laparoscopic surgery was by far the preferred surgical approach after an ICR in CD amongst CSSANZ surgeons at 93% (*n* = 125) (Fig. 3a), which is in keeping with the CD treatment guidelines by ECCO [8]. Laparoscopic surgery has been demonstrated to be superior in reducing post-surgical complications, especially incisional hernias and length of hospital stay, provided adequate expertise is available [17, 18].

The preferred anastomotic configuration was the stapled STSA, preferred by 47% of respondents (Fig. 1). It remained the leading preference even after accounting for surgeon membership status (Fig. 2) and whether the surgeon’s primary facility had a dedicated IBD service (Table 2). The literature supports the preference for stapled STSA. Established findings by He et al. (2014) and subsequent meta-analyses have consistently demonstrated the superiority of the stapled STSA in reducing AL rates (OR 0.45, 95% CI 0.20–1.00) as well as lower clinical recurrence of CD postoperatively (OR 0.20, 95% CI 0.07–0.55) [7]. The favour for the stapled STSA over the ETEA was reflected in the 2019 ECCO CD Treatment guidelines [8], which kept with the observed preference amongst responding surgeons (46 vs 19%). Yet, no single anastomotic technique and configuration are clearly dominant amongst ANZ surgeons (Fig. 1), especially compared to the resounding consensus demonstrated towards the choice of surgical approach and extraction site: 93% laparoscopic, 80% midline peri-umbilical.

The preferred approach to mesenteric resection by 75% of respondents was to resect at the proximal junction of abnormal mesentery, while only 10% performed a radical resection of the mesentery. The literature is conflicted in this regard. The 2016 review by Coffey et al. discussed the role of the mesentery as a potential driver of CD pathophysiology and recurrence, recommending the radical mesenteric resection [9]. Coffey et al. goes on to demonstrate in a 2018 combined cohort study that division of the mesentery in ICR reduces surgical recurrence of CD from 40 to 2.9% (*p = *0.003) when compared to historic resections performed flush along the intestine [11]. This close intestinal approach was preferred by 15% of colorectal surgeons surveyed in ANZ. Coffey et al. (2016) ascribe the enduring conservatism of mesenteric resection in CD to both a historically poor understanding of mesenteric anatomy as well as the technical difficulties of operating in the often-hostile abdomen of advanced Crohn’s disease, acknowledging that mesenteric resection is a more radical surgery.

By contrast, Kono, et al. (2011) recommends preservation of the mesentery but with exclusion of the anastomosis from it, pioneering such an anastomotic configuration in 2003 [15]. In producing a handsewn ETEA isolated from the mesentery, the Kono S anastomosis appears effective in preventing surgical recurrence in 98.6% of patients at 5 and 10 years in a 2016 international multicentre retrospective cohort study by Kono et al. [14]. Further, the Lugio et al*.*(2020) SuPREMe-CD Study, the first RCT to compare the Kono S anastomosis with the conventional stapled STSA recommended by the ECCO, demonstrated a significantly reduced 6-month ePOR ≥ i2 (22.2 vs 62.8%; *p *< 0.001, OR 5.91) and clinical recurrence (8 vs 30.2%; *p* = 0.04, OR 3.47) at 24 months in the Kono S group with no differences in post-operative complications [12]. Kono S adoption amongst ANZ survey respondents was 21% (29), with 15% (21) of respondents preferring anastomosis for ICR in CD. Though not statistically significant, a higher percentage of fellows had performed the Kono S (30 vs 20%; *p = *0.31), suggesting a trend towards newer techniques amongst younger practitioners. Moreover, surgeons at centres with a dedicated IBD service have slightly more uptake (26 vs 11%), potentially suggesting higher Kono S adoption in centres with a larger volume of CD ICR per surgeon; mean diff. 3.82. Likewise, there is a higher percentage of adoption of the Kono S in NSW (39%, 14 of 36) and VIC (25%, 7 of 28), areas with a greater number of metropolitan respondents (Table. 1), who have a higher case load of CD resections compared to their regional counterparts (7.22 vs 4.16; *p = *0.02).

A systematic review by Alshantti et al. (2021) again asserts both the safety and efficacy of the Kono S anastomosis as surgical prophylaxis of both endoscopic and surgical recurrence (0–3.4% vs 15–24.4%) but notes that preservation of the mesentery could negatively influence recurrence rates and argued for further studies to evaluate the impact of mesenteric resection [10]. In view of the continued recognition of the affected mesentery as a potential nidus of recurrence by Alshantti et al. (2021), Holubar et al. (2022) compared the standard Kono S with a variant that includes extended mesenteric excision [10, 16]. This approach of mesenteric excision and exclusion (MEE) was evaluated in a retrospective single-centre cohort study and found to be technically feasible and safe at short-term follow-up [19]. However, the efficacy of the MEE in reducing POR was not studied, of note given that the present survey shows 82% (24 of 29) of CSSANZ surgeons who had performed the Kono S preferred some degree of mesenteric resection. Even amongst respondents who preferred the Kono S, only 19% (4 of 21) prefer to resect close to the intestine as described by Kono et al. (2011), constituting a technical deviation from the current literature, which only demonstrates a reduction in ePOR with the Kono S when implemented with mesenteric preservation [12, 14, 15].

Research abstracts presented at the ECCO meeting in Stockholm, Sweden, in February 2024 provide further relevant information to consider but were unavailable at the time of the survey. Preliminary results from the New York RCT of the Kono S anastomosis included a much larger cohort of 288 than the Luglio et al*.*(2020) study and have shown no difference in ePOR compared to the side-to-side functional end anastomosis using the more granular modified Rutgeerts score (*p* = 0.883) [12, 20]. The SPICY trial group have shown no difference in ePOR between the mesenteric resection group vs mesenteric preservation and employing the STSA in both arms (*p* = 1.0) [21]. These new findings suggest that there is no benefit to the Kono S nor mesenteric resection for ePOR.

Notably, the justification for conducting an ANZ RCT of the Kono S anastomosis becomes apparent [22]. There is a need to generate robust evidence to define the paradigm of mesenteric resection in CD and ultimately support evidence-based surgical decision-making, which may narrow the range of anastomotic approaches across ANZ. As the present survey observed that the majority of CSSANZ surgeons (85%, *n* = 115) prefer including some portion of the mesentery with their ICRs, future trials in ANZ could study the modified Kono S technique with mesenteric resection as an intervention group. The stapled STSA is most widely employed (Fig. 1) and has the most evidence to serve as the control arm [22].

This study is the first survey evaluating approaches of current CSSANZ surgeons in their surgical management of Crohn’s disease. Survey research has been shown to be effective and appropriately rigorous in evaluating subject matters otherwise difficult to study, including current practices [23]. Distribution was assisted by the society for representative and de-identified sampling.

There are several limitations to this study. Survey response rate was low, at 39.9% but in a range of typical study response rates in CSSANZ surveys (52.5 ± 18.3)[23]. The survey used was not validated but reported current practices and will inform directions of future research. Another potential source for error lies in inconsistent survey completion. Four incomplete surveys were submitted, and 8 respondents selected two or more responses to a single question. These responses were included, and where responses exceeded the total number of respondents, percentages were calculated for the number of responses for that survey item rather than the number of respondents. A further consideration is that responses submitted under “other” may potentially fall under listed options, skewing the results away from potentially more dominant practices. This mainly pertains to item G, regarding preferred bowel preparation (Appendix 1), where 30% of respondents selected “other”. No respondent selections were altered to conform to pre-existing options.

A limiting factor to the study of CD is the high degree of heterogeneity inherent in IBD cohorts [24–26]. Consequently, approaches to the surgical management of CD are individually tailored and differ widely. As such, results from CD research may be contrary to expectations but may be overcome by randomisation to control for patient heterogeneity between cohorts in prospective interventional trials [27].

## Conclusion

Among participating colorectal surgeons practicing in Australia and NZ, there was clear consensus regarding surgical approach, where the dominant practice was laparoscopy with a midline peri-umbilical extraction site. Similarly, most respondents reported some degree of mesenteric resection either at the proximal extent of the disease or radically with the intestinal resection. However, anastomotic configuration and technique were domains of resection in CD lacking consensus despite clear guidelines, highlighting an area requiring further research attention.

## Supplementary Information

Below is the link to the electronic supplementary material.

Supplementary file1 (DOCX 16 KB)

## Data Availability

No datasets were generated or analysed during the current study.
