# Supplementary material for: Current approaches to the surgical management of Crohn’s disease in Australia and New Zealand
Source: Int J Colorectal Dis. 2025 Jan 3;40(1):4. doi: 10.1007/s00384-024-04778-6 (PMC11698757; doi:10.1007/s00384-024-04778-6)
Supplement: Supplementary file 1 — Supplementary file1 (DOCX 16 KB) [file 384_2024_4778_MOESM1_ESM.docx]

**APPENDIX 1**

CSSANZ Survey Questions

1. Demographics

Age

Sex

Fellow/ consultant

State/NZ

Years post fellowship

Primary facility: Metropolitan/regional

Approximate number of ileocolic resections for Crohn’s disease per year

Do you have a dedicated IBD service at your primary hospital? Y/N

1. Preferred anastomotic configuration

Side to side (stapled/handsewn)

End to end

End to side (stapled/handsewn)

Isoperistaltic side to side (stapled/handsewn)

Kono S

Other

1. Preferred approach

Laparoscopic

Open

Robotic

SILS

Other

1. Preferred extraction site (if minimally invasive approach)

Midline infra-umbilical

Midline peri-umbilical

Pfannenstiel

Other

1. How do you address the mesentery?

Resect at proximal junction of abnormal mesentery

Close intestinal resection through the abnormal thickened mesentery

Radical resection of mesentery

1. What margin of normal tissue do you aim for? (…cms)
2. Bowel preparation

Full bowel prep

Clear fluids only

Other … expand

1. Have you performed the Kono S anastomosis? (Y/N)

If yes, how many cases?

1. If already performing the Kono S anastomosis, are you studying outcomes in:

An RCT

Prospective audit

Retrospective cohort analysis
